# Mast Cells Exhibiting Strong Cytoplasmic Staining for IgE and High Affinity IgE Receptor are Increased in IgG4-Related Disease

**DOI:** 10.1038/s41598-018-23043-9

**Published:** 2018-03-15

**Authors:** Kenji Nishida, Yuka Gion, Mai Takeuchi, Takehiro Tanaka, Tatsuki R. Kataoka, Tadashi Yoshino, Yasuharu Sato

**Affiliations:** 1 Department of Pathology, Okayama University Graduate School of Medicine, Dentistry and Pharmaceutical Sciences, Okayama, Japan; 2 Department of Pathology, Okayama University Hospital, Okayama, Japan; 3 Department of Diagnostic Pathology, Kyoto University Hospital, Kyoto, Japan; 4 Division of Pathophysiology, Okayama University Graduate School of Health Sciences, Okayama, Japan

## Abstract

Immunoglobulin G4 (IgG4)-related disease is characterized by elevated serum IgG4 levels and increased numbers of IgG4-positive cells. However, its pathogenesis is not fully understood. We previously suggested that mast cells may play an important role in IgG4-related disease. In this study, we confirmed the characteristics of mast cells in IgG4-related lymphadenopathy by using immunohistochemistry and dual immunofluorescence. We analyzed 23 cases of IgG4-related lymphadenopathy and compared them with 23 cases of non-specific lymphoid hyperplasia. The majority of patients with IgG4-related lymphadenopathy had cervical lesions with involvement of other organs. Immunohistologically, mast cells with strong cytoplasmic staining for immunoglobulin E and high affinity immunoglobulin E receptor were significantly increased in IgG4-related lymphadenopathy as compared to those in non-specific lymphoid hyperplasia (mean: 3.83 ± 3.99 cells per high power field and 7.14 ± 8.21 cells per high power field, respectively; *P* = 0.007 and *P* = 0.011). In addition, dual immunofluorescence assay showed that immunoglobulin E and high affinity immunoglobulin E receptor staining exhibited a cytoplasmic granular pattern in IgG4-related lymphadenopathy, suggesting internalization of the antibodies and receptors. Our findings showed that mast cell activation might be involved in the pathogenesis of IgG4-related disease.

## Introduction

Immunoglobulin G4-related disease (IgG4-RD) was initially described in 2001 as sclerosing pancreatitis with elevated serum IgG4 levels^1^, and has subsequently been described in various organ systems, including the biliary tree, salivary glands, periorbital tissues, kidneys, lungs, meninges, aorta, breast, prostate, thyroid gland, pericardium, skin, and lymph nodes^2^. This is a fibro-inflammatory condition characterized by a tendency to form tumefactive lesions at multiple sites, with a distinctive histopathological appearance, and often, but not always, elevated serum IgG4 levels^2,3^. Although glucocorticoids are effective and the standard therapy for IgG4-RD, the disease is known to be likely to relapse during tapering or cessation of the medicine. Yamamoto *et al*.^4^ reported that more than half of patients with Mikulicz’s disease experience relapse during steroid therapy. Thus, IgG4-RD has been deemed incurable in Japan, and investigators have attempted to elucidate its pathogenesis within the last decade.

There are many hypotheses regarding the pathogenesis of IgG4-RD that involve autoimmunity, molecular immunity, specific genotypes, intestinal bacterial flora, allergies, and other factors, which are yet to be proven. From a histopathological point of view, upregulation of T helper 2 (Th2) (interleukin [IL]-4, IL-5, and IL-13) and regulatory T (Treg) (IL-10 and transforming growth factor beta 1) cytokines was detected in affected tissues of patients with autoimmune pancreatocholangitis^5^, suggesting that the immune reaction mediated by these cytokines may be responsible for the lesions. These findings have also been confirmed in salivary glands, ocular adnexa, and kidneys^6–8^. However, to the best of our knowledge, only a few previous reports have investigated the mechanism underlying Th2 and Treg activation in IgG4-RD.

We previously reported a significant upregulation of IL-4, IL-10, IL-13, and transforming growth factor beta 1 in IgG4-related submandibular gland disease^9^. These findings in IgG4-related sialoadenitis were consistent with those of other previous reports^6,10,11^. In addition, we discerned that the infiltrating cells expressing Th2 and Treg cytokines were histomorphologically similar to mast cells and exhibited strong cytoplasmic staining for immunoglobulin E (IgE). Interestingly, although the number of c-kit-positive mast cells was not significantly increased in IgG4-related submandibular gland disease as compared to that in sialolithiasis and normal submandibular glands, the number of strongly cytoplasmic IgE-positive mast cells was significantly increased in IgG4-related submandibular gland disease. Based on these findings, we note mast cells exhibiting strong cytoplasmic staining for IgE as a key role in IgG4-RD. However, the underlying mechanisms and immunological significance of strong cytoplasmic IgE staining remain unclear.

In this study, we aimed to confirm whether mast cells exhibiting strong cytoplasmic staining for IgE were noted in IgG4-related lymphadenopathy cases and to identify the significance of the strong cytoplasmic staining.

## Materials and Methods

### Patients and samples

Tissue samples were obtained from 23 cases of IgG4-related lymphadenopathy. Tissue samples from an equal number of cases (*n* = 23) of non-specific lymphoid hyperplasia were used as disease controls. All slides were jointly reviewed by K.N., M.T., and Y.S. Formalin-fixed paraffin-embedded specimens were used for immunohistochemistry and dual immunofluorescence. All tissue samples were obtained with the approval of the Institutional Review Board of Okayama University (Okayama, Japan). Informed consent for the use of their samples in research was obtained from all patients.

### Histological examination and immunohistochemistry

The following methods were carried out in accordance with approved guidelines. All experimental protocols were approved by the Institutional Review Board at Okayama University.

Excisional biopsies were obtained from lymph node lesions in 23 patients with IgG4-related lymphadenopathy and an equal number of patients (*n* = 23) with non-specific lymphoid hyperplasia. The specimens were fixed in 10.0% formaldehyde and embedded in paraffin. Serial 4-µm-thick sections were cut from the paraffin-embedded tissue blocks and stained with hematoxylin and eosin. Sections were immunohistochemically stained using an automated Bond Max stainer (Leica Biosystems; Melbourne, Australia). The following primary antibodies were used: polyclonal IgG (dilution 1:20,000; Dako, Glostrup, Denmark), IgG4 (HP6025, dilution 1:10,000; The Binding Site, Birmingham, UK), KIT/CD117 (A4502, dilution 1:600; Dako), IgE (A094, dilution 1:500; Dako), and high affinity IgE receptor (FcεRI) (Ab54411, dilution 1:500; Abcam, Cambridge, UK). Following immunohistochemical staining, the number of IgG4- and IgG-positive cells were estimated in areas with the highest density of IgG4-positive cells. In accordance with the consensus statement on the pathological features of IgG4-RD^3^, three different high power fields (HPFs) (eyepiece: ×10, lens: ×40) were examined to calculate the average number of IgG4-positive cells per HPF and the IgG4-positive/IgG-positive cell ratio. IgE- and FcεRI-positive cells were counted in three different HPFs (eyepiece: ×10, lens: ×20) that were determined to have the highest density of positive cells. The average number of positive cells per HPF was calculated.

### Dual immunofluorescence assays

For indirect dual immunofluorescence assays, paraffin sections were stained with the primary antibodies for IgE and FcεRI or FcεRI and c-kit. Fluorescein isothiocyanate-conjugated secondary antibodies (Alexa Fluor anti-mouse 555 and Alexa Fluor anti-rabbit 488; both from Life Technologies, Carlsbad, CA, USA) were used at a dilution of 1:400. The stained specimens were examined using a conventional immunofluorescence microscope (IX71; Olympus, Tokyo, Japan).

### Statistical analysis

Data are presented as the means and standard deviations. All statistical analyses were performed using the Mann-Whitney *U* test in the Statistical Package for the Social Sciences for Windows, software version 14.0 (SPSS Inc., Chicago, IL, USA). A *P* < 0.05 was considered statistically significant.

## Results

### Histological confirmation of IgG4-related lymphadenopathy

The clinicopathological characteristics of the patients with IgG4-related lymphadenopathy are summarized in Table 1. The cohort was comprised of 14 men and 9 women, with a median age of 61 (range, 45–82) years. Post-biopsy serum IgG or IgG4 levels were obtained for all patients except for Patient 23. On initial clinical examination, 14 patients presented with localized cervical lymphadenopathy. Seven patients had additional areas of lymphadenopathy (≥2) and 9 patients had extranodal lesions (e.g., in the salivary and lacrimal glands). All patients were followed up with regular imaging, laboratory assays, and clinical examinations. The median follow-up period was 42 (range, 2–154) months. During follow-up, 10 patients (43%) exhibited relapse in the residual lymph nodes or their disease had progressed to the development of other lymph nodes or extranodal lesions, including those of the submandibular and lacrimal glands. The average number of IgG- and IgG4-positive cells was 224.1 (range: 85.7–378) and 259.1 (range: 97.7–437.3) per HPF, respectively. In all cases, the IgG4-positive/IgG-positive cell ratios were >60.0%. The average numbers of IgE and FcεRI strongly cytoplasmic positive cells were 3.83 (range: 85.7–378) and 8.50 (range: 97.7–437.3) per HPF, respectively.

**Table 1:** Histological and serological findings of patients with IgG4-related lymphadenopathy.

Case No.	Age	Sex	LN site of biopsy	LN size (mm)	Location	Extranodal lesion	Allergy	Relapse	IgG4+ cells (/HPF)	IgG+ cells (/HPF)	Histologically IgG4+/IgG+ cell ratio (%)	IgE-count (HPF)	FcεRI-count (/HPF)	Serum-IgG4 (mg/dL)	Serum-IgG (mg/dl)
1	50	M	Axillary	21	Bilateral cervical, left axillary, paraaortic, mesenteric	—	Pollenosis, rhinitis	Yes	277	308.3	91.45	0.33	0.67	158*	1930*
2	66	M	Cervical	16	Bilateral cervical	—	None	Yes	235	266.3	87.5	4.33	5.33	89*	1511*
3	50	F	Cervical	15	Bilateral cervical, right infraclavicular	—	None	No	218	202	112.2	0.33	0	24*	n.e.
4	45	F	Cervical	21	Left cervical	—	Bronchilal asthma	No	186	212.3	91.14	0.33	1.33	18.3*	1202*
5	73	F	Abdominal	30	Paraaortic, mesenteric	—	Drug	No	85.7	97.7	90.05	1	1.67	16.5*	1206
6	51	F	Cervical	21	Bilateral cervical	—	Asthma	Yes	261	262.3	99.16	0.67	5.67	223*	1425
7	50	M	Cervical	23	Right cervical	—	Allergic rhinitis	Yes	245	295.3	81.98	3	5	17.8*	1072*
8	46	M	Cervical	13	Left cervical	—	None	No	241	279.3	86.42	0	13.67	94.7*	1505*
9	71	M	Cervical	18	Right cervical	—	Bronchilal asthma	No	378	258	152.2	1.67	1.67	58.7*	644*
10	67	M	Cervical	19	Bilateral cervical, hilar	Right lung	Allergic rhinitis	Yes	218	297.7	73.83	5.33	10.33	389*	1619*
11	58	F	Cervical	18	Right cervical	Bilateral parotid glands, left lobe of thyroid gland	Pollenosis	No	188	282	78.76	3.67	33.72	51*	1297*
12	61	M	Cervical	32	Bilateral cervical	—	None	Yes	364	437.3	83.48	3	13	650*	2200*
13	72	M	Cervical	20	Left cervical, mediastinal	Retroperitoneal fiborosis, sclerosing cholangitis, pituitary gland	Asthma	Yes	230	339	68.74	13.33	23.67	1750*	n.e.
14	70	M	Cervical	10	Bilateral cervical	Parotid gland, bilateral lungs	None	No	173	159.7	108.9	5.67	4.33	236*	1466*
15	57	F	Cervical	6	Left cervical, bilateral axillary, bilateral infraclavicular	—	None	Yes	219	346.3	65.02	1	0	12.7*	1542*
16	82	F	Cervical	28	Bilateral cervical, bilateral axillary, hilar	Bilateral submandibular glands, bilateral lacrimal glands, orbit, right lung	None	Yes	267	283	94.43	1	1.33	n.e.	4057*
17	75	F	Cervical	27	Left cervical	—	Asthma	No	199	272.3	78.1	7	11.67	88.9*	n.e.
18	49	M	Cervical	28	Left cervical	Left submandibular gland	None	No	120	167	72.58	3.67	9	98.2*	1000*
19	72	M	Cervical	25	Bilateral cervical	—	Drug	No	220	278.3	79.13	8.67	11.67	71.2*	1201.7*
20	66	M	Cervical	12	Right cervical	Right submandibular gland, left lobe of thyroid gland	None	Yes	251	316.7	81.47	1.67	2.67	83.3*	1188.6*
21	57	F	Cervical	24	Bilateral cervical, hilar	Bilateral submandibular glands, bilateral lacrimal glands	Asthma	No	218	204	110.9	4	2.67	630	2011
22	54	M	Axillary	35	Right axillary	Right lung	Pollenosis, bronchial asthma	No	178	161.3	110.8	3.33	2.67	45*	900*
23	72	M	Cervical	24	Cervical	—	None	No	182	234	78.48	15	33.667	n.e.	n.e.

M; male, F; female, LN; lymph node, n.e.; not examined. *; measurement of serum levels after biopsy.

**Figure 1:**
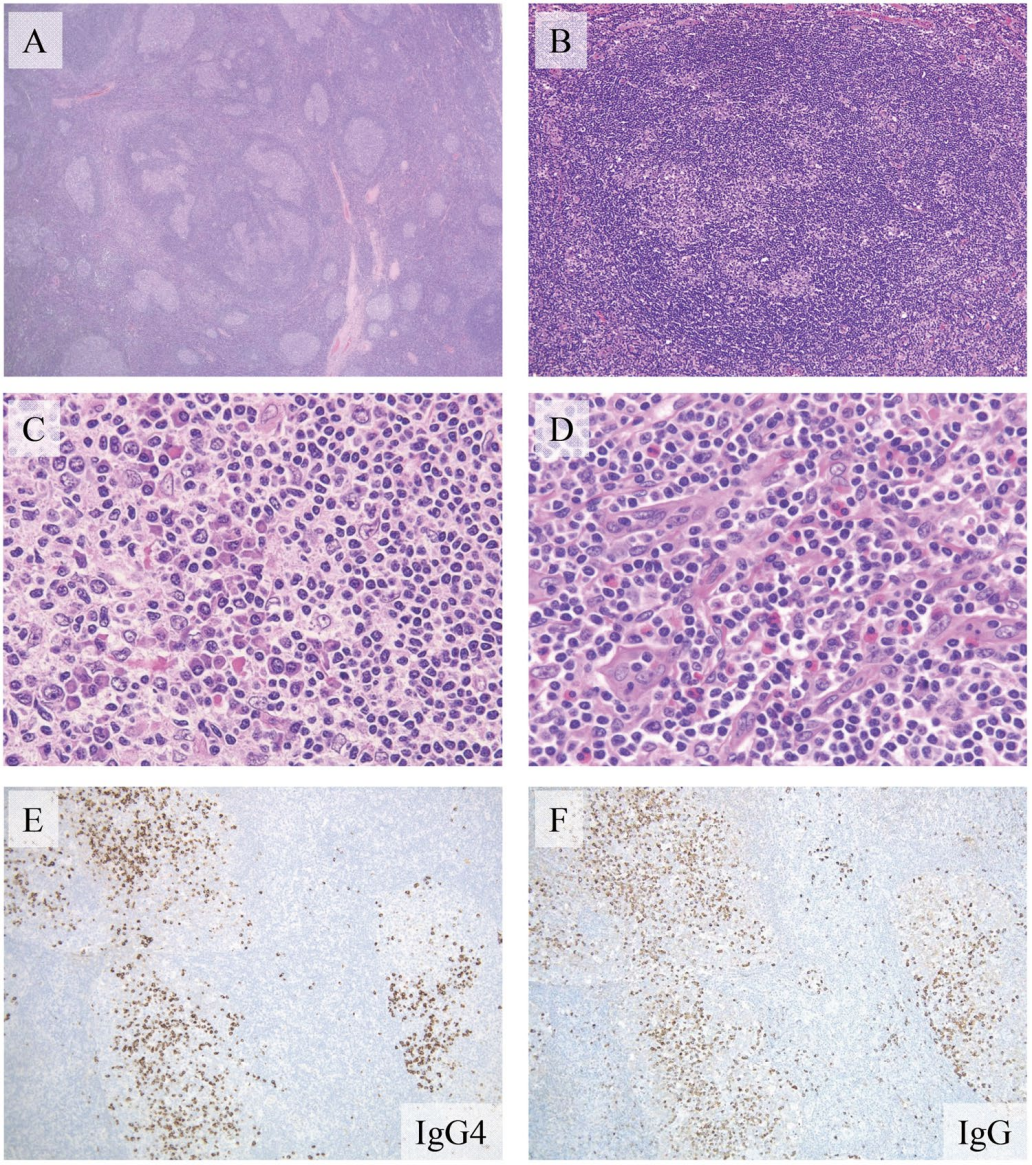
Histological features of immunoglobulin G4 (IgG4)-related lymphadenopathy (Patient 19). (**A**) Lymph nodes showing multiple follicular hyperplasia with progressively transformed germinal centers (hematoxylin and eosin [H&E] staining, 20× magnification). (**B**) Irregular structure of a germinal center owing to the infiltration of small lymphocytes from the mantle zone (H&E staining, 100× magnification). (**C**) Abundant plasma cells infiltrating the germinal centers (H&E staining, 200× magnification). (**D**) Many eosinophils infiltrating the interfollicular zone (H&E staining, 200× magnification). (**E**,**F**) The majority of IgG4-positive plasma cells are localized in the germinal centers. The IgG4-positive/IgG-positive plasma cell ratio is >70% (IgG4 and IgG immunostaining, 100× magnification).

We confirmed that the tissue specimens from all 23 cases with IgG4-RD showed typical histological features of progressively transformed germinal center (PTGC)-type IgG4-related lymphadenopathy (Fig. 1A–F).

### Histological features of non-specific lymphoid hyperplasia

The specimens exhibited multiple lymphoid follicle formations with moderate lymphocytic infiltration of interfollicular areas. Compared to the histological features of IgG4-related lymphadenopathy, those of non-specific lymphoid hyperplasia showed no PTGC, and less infiltration of plasma cells and/or eosinophils. Dermatopathic and autoimmune disease-associated lymphadenopathies, including rheumatoid arthritis, systemic lupus erythematosus, and Sjögren’s syndrome, were excluded.

### Clinical summary

The clinical differences between IgG4-related lymphadenopathy patients and those with non-specific lymphoid hyperplasia are shown in Table 2. The median age was 61 (range, 45–82) years for IgG4-related lymphadenopathy and 65 (range, 18–81) for non-specific lymphoid hyperplasia (*P* = 0.369). The overall median age was 63 (range, 18–82). There was no significant difference in sex between the two groups (IgG4-related lymphadenopathy: 14 men and 9 women, non-specific lymphoid hyperplasia: 12 men and 11 women; *P* = 0.562). Patients with IgG4-related lymphadenopathy had larger lymph nodes than patients with non-specific lymphoid hyperplasia (mean length, 21.1 [range, 6.0–35.0] *vs*. 12.3 [range, 5.0–26.0] mm, respectively; *P* < 0.001). Lymphadenopathy occurred in the cervical region in 21 patients with IgG4-related lymphadenopathy and in 10 patients with non-specific lymphoid hyperplasia (91.3% *vs*. 43.5%, respectively; *P* < 0.001).

**Table 2:** Clinical characteristics of IgG4-related lymphadenopathy and non-specific lymphoid hyperplasia.

	IgG4-related lymphadenopathy	Non-specific lymphoid hyperplasia	*P*
Number	23	23	—
Age, years	61.5 (45–82)	57.3 (15–81)	0.37
Gender; male (%)	14 (60.9%)	12 (52.2%)	0.56
Size of lymph node (mm)	21.1 (6–35)	12.3 (5–26)	0.000025
Site of lymph node			
Cervical	21 (91.3%)	10 (43.5%)	0.00038
Axillary	4 (17.4%)	6 (26.1%)	0.49
Supraclavicular	3 (13.0%)	1 (0.043%)	0.31
Hilar	4 (17.4%)	1 (0.043%)	0.16
Inguinal	0 (0%)	5 (21.7%)	0.022
Multiple	8 (34.8%)	6 (26.1%)	0.53
Extranodal lesion	9 (39.1%)	—	—
	(salivary gland, lacrimal gland, lung, etc.)		

### IgE and FcεRI staining patterns in IgG4-related lymphadenopathy

**Figure 2:**
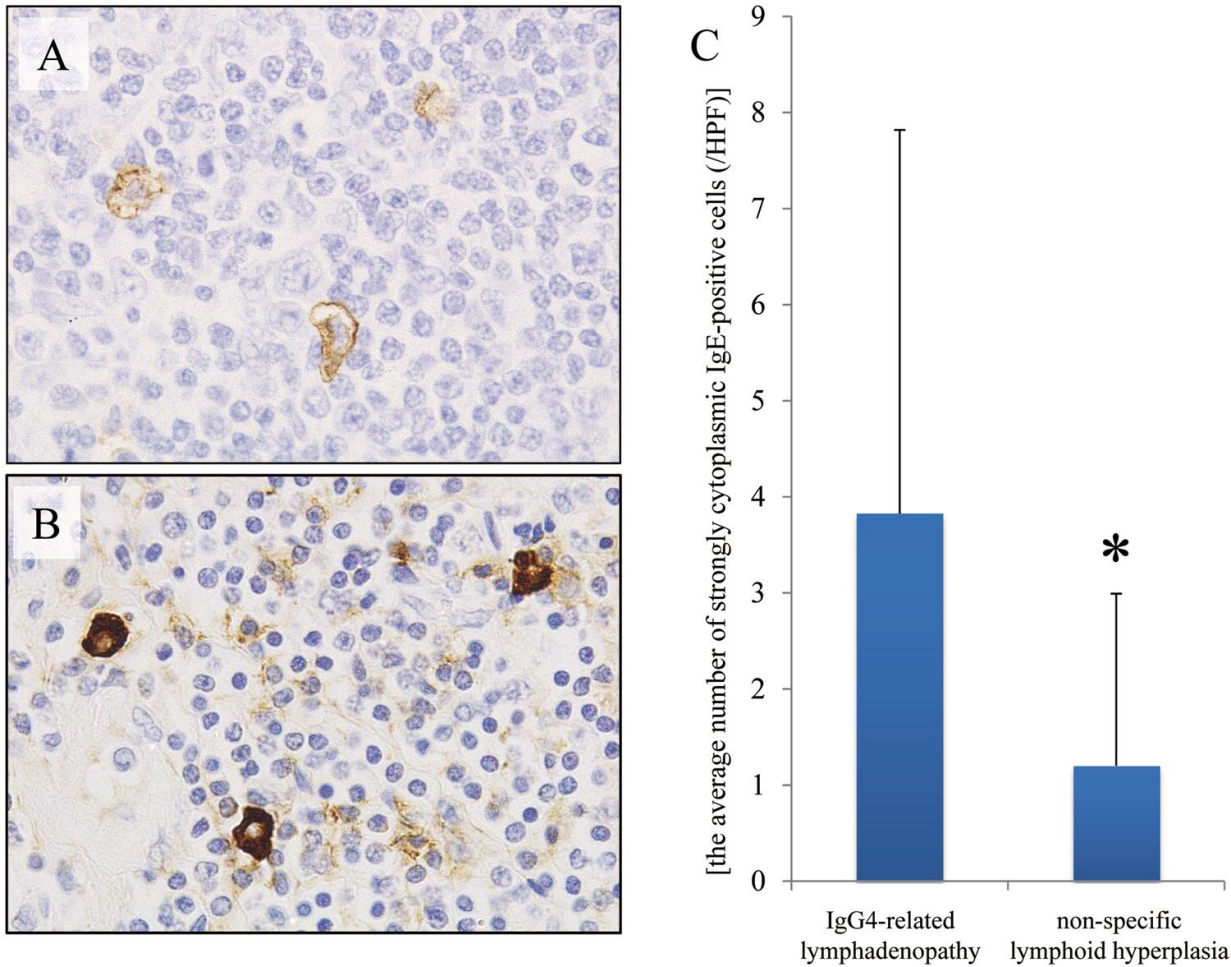
Staining for immunoglobulin E (IgE). (**A**) Mast cells exhibiting moderate-to-week IgE staining and a membranous localization in non-specific lymphoid hyperplasia. (**B**) In contrast, mast cells exhibit strong cytoplasmic staining for IgE in immunoglobulin G4-related lymphadenopathy (Patient 19). (**C**) The number of strongly cytoplasmic IgE-positive cells is counted in each high power field and was significantly larger in immunoglobulin G4-related lymphadenopathy than in non-specific lymphoid hyperplasia (^*^P < 0.01).

**Figure 3:**
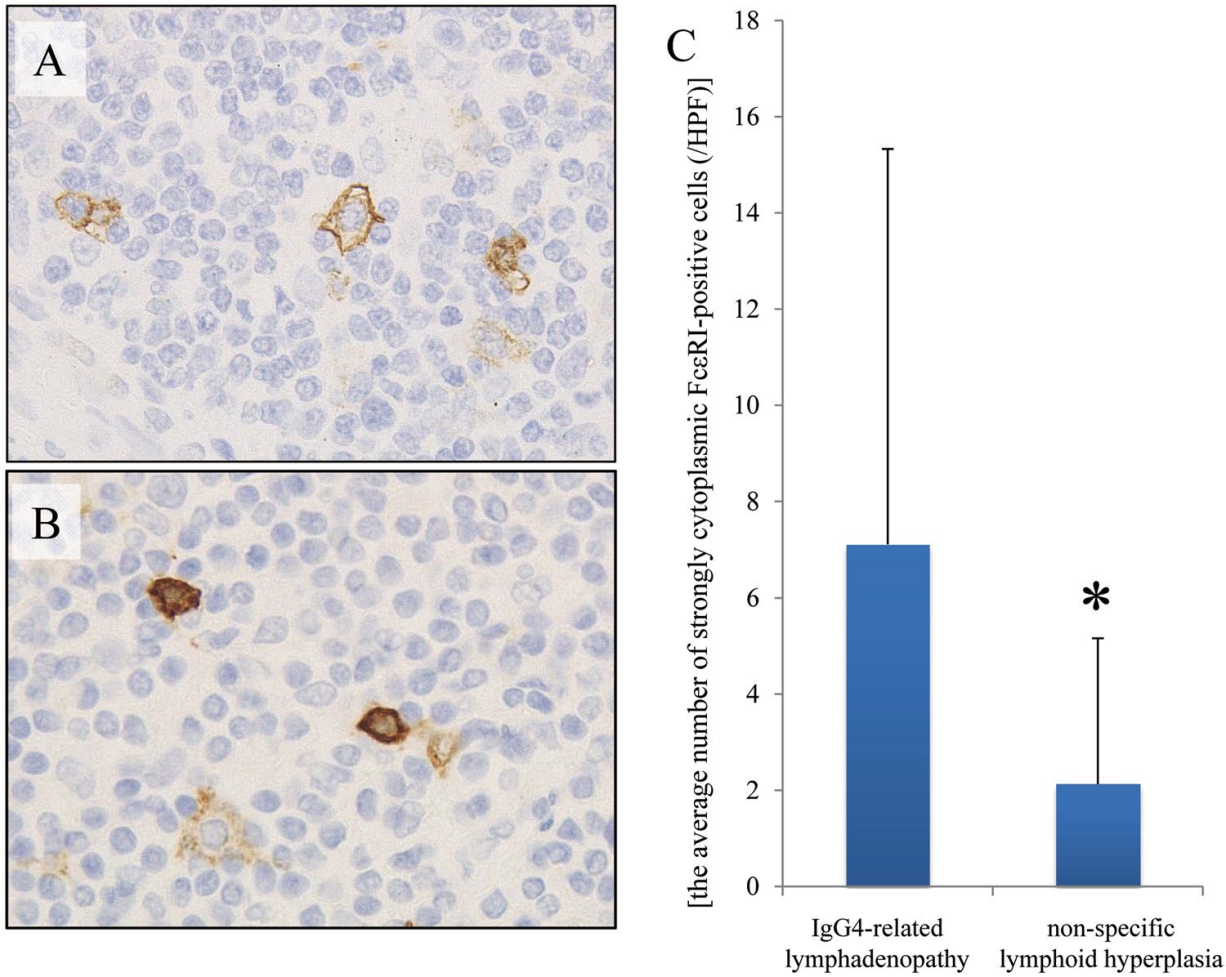
Staining pattern of high affinity immunoglobulin E (IgE) receptor (FcεRI) is similar to that of IgE. (**A**) Infiltrating mast cells in non-specific lymphoid hyperplasia exhibit weak membranous FcεRI staining. (**B**) In contrast, infiltrating mast cells in immunoglobulin G4-related lymphadenopathy exhibit strong cytoplasmic staining (Patient 19). (**C**) Strongly cytoplasmic FcεRI-positive cells are significantly increased in immunoglobulin G4-related lymphadenopathy as compared to those in non-specific lymphoid hyperplasia (^*^P = 0.01).

A large number of IgE- and FcεRI-positive cells were detected in the tissues of patients with IgG4-related lymphadenopathy and non-specific lymphoid hyperplasia (Supplementary Figures 1 and 2). In the non-specific lymphoid hyperplasia specimens, most mast cells stained for IgE and FcεRI with a surface membrane pattern (Figs 2A and 3A). In contrast, in the IgG4-related lymphadenopathy specimens, some mast cells stained for IgE and FcεRI with a strongly cytoplasmic pattern (Figs 2B and 3B). There was a greater number of strongly cytoplasmic IgE-positive cells in the IgG4-related lymphadenopathy specimens than in the non-specific lymphoid hyperplasia specimens (mean: 3.83 ± 3.99 *vs*. 1.20 ± 1.79 cells per HPF, respectively; *P* = 0.007) (Fig. 2C). Strongly cytoplasmic FcεRI-positive cells were also detected more frequently in the IgG4-related lymphadenopathy specimens than in the non-specific lymphoid hyperplasia specimens (mean: 7.14 ± 8.21 *vs*. 2.13 ± 3.03 cells per HPF, respectively; *P* = 0.011) (Fig. 3C).

### Dual immunofluorescence assay

**Figure 4:**
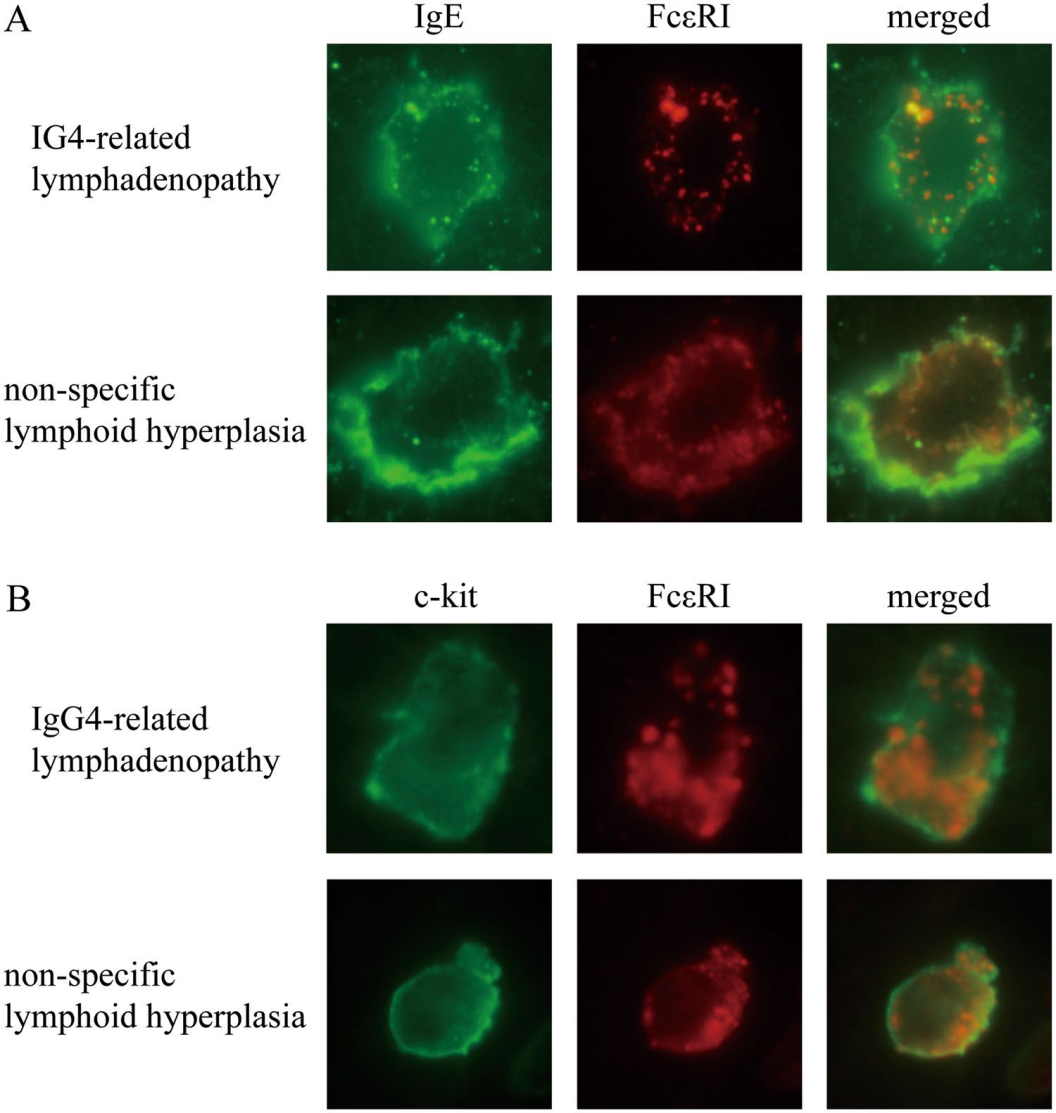
Dual immunofluorescence of mast cells in immunoglobulin G4-related lymphadenopathy and non-specific lymphoid hyperplasia. (**A**) In immunoglobulin G4-related lymphadenopathy, both immunoglobulin E (IgE) and high affinity IgE receptor (FcεRI) exhibit a granular pattern, some of which overlap, resulting in yellow spots. In non-specific lymphoid hyperplasia, IgE stains the surface membrane, and FcεRI does not exhibit a granular pattern. (**B**) Staining for c-kit and FcεRI.

In the IgG4-related lymphadenopathy specimens, IgE and FcεRI staining exhibited a cytoplasmic granular pattern (Fig. 4A,B). Intracytoplasmic granules stained positive for IgE (green) and FcεRI (red), some of which overlapped as yellow spots. In the non-specific lymphoid hyperplasia specimens, IgE and FcεRI stained the surface membrane. These cells were positive for c-kit.

## Discussion

Although many previous studies regarding IgG4-RD have been reported in the last decade, the pathogenesis of the disease remains unclear. Recent studies have reported that IgG4-RD is a Th2- and Treg-dominant disease, and the activation of these cytokines may be responsible for this disease^5,6,9,10,12–15^. However, studies investigating the mechanism underlying cytokine activation in IgG4-RD are few. In this study, we focused on mast cells exhibiting strong cytoplasmic staining for IgE.

Comprehensive diagnostic criteria consisted of elevated serum IgG4 concentrations (>135 mg/dl), but not in all cases^3,16,17^. Some patients with PTGC-type IgG4-related lymphadenopathy have normal serum IgG4 levels when measurements are obtained after biopsy, at which time there are no residual primary lesions^18^. In our current data, serum IgG4 levels in 16 cases were <135 mg/dl. Furthermore, the recurrence ratio of IgG4-RD has been reported at 21.3%^17^. Some patients with PTGC-type IgG4-related lymphadenopathy have also experienced either persistence or relapse of residual lymph nodes^18^. In this study, 10 of 23 patients progressed to the development of other lymph nodes or extranodal lesions with elevated levels of serum IgG4.

Here, we confirmed that mast cells exhibiting strong cytoplasmic staining for IgE, were increased in IgG4-related lymphadenopathy. This is consistent with our previous report of submandibular gland disease^9^. Regarding the significance of strong cytoplasmic staining for IgE, we posit two hypotheses: overstaining and endocytosis.

IgG4-RD is frequently complicated by allergic conditions^19,20^. We previously reported that 52% of patients with IgG4-related lymphadenopathy also had allergic disease^18^. Our current study showed that 15 of 19 (78.9%) patients examined had allergic conditions. Furthermore, elevation of serum IgE levels, defined as IgE >250 IU/ml, was observed in more than half of patients with IgG4-RD^17^, suggesting that strong cytoplasmic staining for IgE might indicate only overstaining. However, this study confirmed that mast cells exhibiting strong cytoplasmic staining for FcεRI also increased, as did staining for IgE.

Recent reports have shown that the activation of mast cells leads to internalization of IgE and FcεRI^21,22^. Accordingly, we added dual immunofluorescence assays, resulting in IgE and FcεRI staining exhibited as multiple cytoplasmic granules, some of which overlapped in IgG4-related lymphadenopathy. In contrast, staining in non-specific lymphoid hyperplasia was localized to the surface membrane. These results showed that the significance of strong cytoplasmic staining for IgE and FcεRI might reflect the internalization of IgE and FcεRI due to mast cell activation in IgG4-RD. Mast cells are activated by various biological substances (e.g., exogenous stimuli, endogenous peptides, chemokines, components of the complement system, and Fc receptors for IgE), leading to degranulation and the production of mediators such as cytokines and eicosanoids^23^. Some previous studies have suggested that response to antigen presentation might be involved in the pathogenesis of IgG4-RD^20,24–26^. Our current study suggests that some antigens might activate mast cells. However, additional assays are needed to elucidate the functional significance of IgE and FcεRI internalization. Although previous reports clarified the internalized FcεRI trafficking in early endosomes^23^, no published reports have investigated the trafficking completely.

Other Th subsets (T follicular helper cells or CD4+ cytotoxic T lymphocytes) and other immune cells (M2 macrophages) have recently been reported to participate in the pathogenesis of IgG4-RD^20,25,27–29^. Mattoo *et al*.^25^ reported prominent clonal expansions of CD4+ SLAMF7+ cytotoxic T lymphocytes but not CD4+GATA3+ memory Th2 cells in patients with IgG4-RD. This strongly suggests that these cytotoxic T lymphocytes expanded in response to a specific causal antigen. Furukawa *et al*.^29^. suggested that M2 macrophages producing IL-33 are deeply involved in the activation of Th2 immune responses in IgG4-RD. Therefore, IgG4-RD might progress via the interconnected network of various Th subsets or immune cells.

In conclusion, we confirmed that mast cells exhibiting strong cytoplasmic staining for IgE and FcεRI were increased in IgG4-related lymphadenopathy, implying the activation of mast cells. Since mast cells “communicate” with various cells to produce a mediator, a better understanding of the role of mast cells could enable us to understand the pathogenesis of IgG4-RD.

## Electronic supplementary material

Supplementary Figure 1-2

## References

[1] Hamano H (2001). High serum IgG4 concentrations in patients with sclerosing pancreatitis. N Engl J Med..

[2] Stone JH (2012). IgG4-related disease. N Engl J Med..

[3] Deshpande V (2012). Consensus statement on the pathology of IgG4-related disease. Mod Pathol..

[4] Yamamoto, M. *et al*. Evaluation and Clinical Validity of a New Questionnaire for Mikulicz’s Disease. *Int J Rheumatol*. 283459, 10.1155/2012/283459 (2012). 10.1155/2012/283459 PMC3357487 22649453

[5] Zen Y (2007). Th2 and regulatory immune reactions are increased in immunoglobin G4-related sclerosing pancreatitis and cholangitis. Hepatology..

[6] Tanaka A (2012). Th2 and regulatory immune reactions contribute to IgG4 production and the initiation of Mikulicz disease. Arthritis Rheum..

[7] Ohno K (2015). A subset of ocular adnexal marginal zone lymphomas may arise in association with IgG4-related disease. Sci. Rep..

[8] Kawamura E (2015). Immunohistological analysis for immunological response and mechanism of interstitial fibrosis in IgG4-related kidney disease. Mod Rheumatol..

[9] Takeuchi M (2014). T helper 2 and regulatory T-cell cytokine production by mast cells: a key factor in the pathogenesis of IgG4-related disease. Mod Pathol..

[10] Maehara T (2012). Interleukin-21 contributes to germinal centre formation and immunoglobulin G4 production in IgG4-related dacryoadenitis and sialoadenitis, so-called Mikulicz’s disease. Ann Rheum Dis..

[11] Tsuboi H (2014). DNA microarray analysis of labial salivary glands in IgG4-related disease: comparison with Sjogren’s syndrome. Arthritis Rheumatol..

[12] Takeuchi M (2015). Interleukin 13-positive mast cells are increased in immunoglobulin G4-related sialadenitis. Sci. Rep..

[13] Tsuboi H (2012). Analysis of IgG4 class switch-related molecules in IgG4-related disease. Arthritis Res Ther..

[14] Miyake K (2008). Peripheral CD4+ T cells showing a Th2 phenotype in a patient with Mikulicz’s disease associated with lymphadenopathy and pleural effusion. Mod Rheumatol..

[15] Kanari H (2010). Role of Th2 cells in IgG4-related lacrimal gland enlargement. Int Arch Allergy Immunol..

[16] Umehara H (2012). Comprehensive diagnostic criteria for IgG4-related disease (IgG4-RD), 2011. Mod Rheumatol..

[17] Yamada K (2017). New clues to the nature of immunoglobulin G4-related disease: a retrospective Japanese multicenter study of baseline clinical features of 334 cases. Arthritis Res Ther..

[18] Sato Y (2012). Association between IgG4-related disease and progressively transformed germinal centers of lymph nodes. Mod Pathol..

[19] Saeki T (2018). Comparison of clinical and laboratory features of patients with and without allergic conditions in IgG4-related disease: A single-center experience in Japan. Mod Rheumatol.

[20] Akiyama M (2016). Enhanced IgG4 production by follicular helper T cells and the involvement of follicular helper 1 T cells in the pathogenesis of IgG4-related disease. Arthritis Res Ther..

[21] Fattakhova GV (2009). Endosomal trafficking of the ligated FceRI receptor. Mol Immunol..

[22] Molfetta R (2005). CIN85 regulates the ligand-dependent endocytosis of the IgE receptor: a new molecular mechanism to dampen mast cell function. J Immunol..

[23] Abraham SN (2010). Mast cell-orchestrated immunity to pathogens. Nat Lev Immunol..

[24] Wallace ZS (2015). Plasmablasts as a biomarker for IgG4-related disease, independent of serum IgG4 concentrations. Ann Rheum..

[25] Mattoo H (2016). Clonal expansion of CD4(+) cytotoxic T lymphocytes in patients with IgG4-related disease. J Allergy Clin Immunol..

[26] Hubers, L. M. *et al*. Annexin A11 is targeted by IgG4 and IgG1 autoantibodies in IgG4-related disease. *Gut*. 1, 10.1136/gutjnl-2017-314548 (2017). 10.1136/gutjnl-2017-314548 28765476

[27] Akiyama M (2015). Number of Circulating Follicular Helper 2 T Cells Correlates With IgG4 and Interleukin-4 Levels and Plasmablast Numbers in IgG4-Related Disease. Arthritis Rheumatol..

[28] Maehara T (2017). Lesional CD4+IFN-γ+ cytotoxic T lymphocytes in IgG4-related dacryoadenitis and sialoadenitis. Ann Rheum Dis..

[29] Fukukawa S (2017). Interleukin-33 produced by M2 macrophages and other immune cells contributes to Th2 immune reaction of IgG4-related disease. Sci Rep..

