# Supplementary material for: Mast Cells Exhibiting Strong Cytoplasmic Staining for IgE and High Affinity IgE Receptor are Increased in IgG4-Related Disease
Source: Sci Rep. 2018 Mar 15;8:4656. doi: 10.1038/s41598-018-23043-9 (PMC5854596; doi:10.1038/s41598-018-23043-9)
Supplement: Supplementary file 1 — Supplementary Figure 1-2 [file 41598_2018_23043_MOESM1_ESM.pdf]

Mast Cells Exhibiting Strong Cytoplasmic Staining for IgE and High Affinity IgE Receptor are Increased in IgG4-Related Disease

Kenji Nishida[1], Yuka Gion[1], Mai Takeuchi[1], Takehiro Tanaka[2], Tatsuki R Kataoka[3], Tadashi Yoshino[1], Yasuharu Sato[1,4]

[1]Department of Pathology, Okayama University Graduate School of Medicine, Dentistry and Pharmaceutical Sciences, Okayama, Japan.
[2]Department of Pathology, Okayama University Hospital, Japan.
[3]Department of Diagnostic Pathology, Kyoto University Hospital, Kyoto.
[4]Division of Pathophysiology, Okayama University Graduate School of Health Sciences, Okayama, Japan.

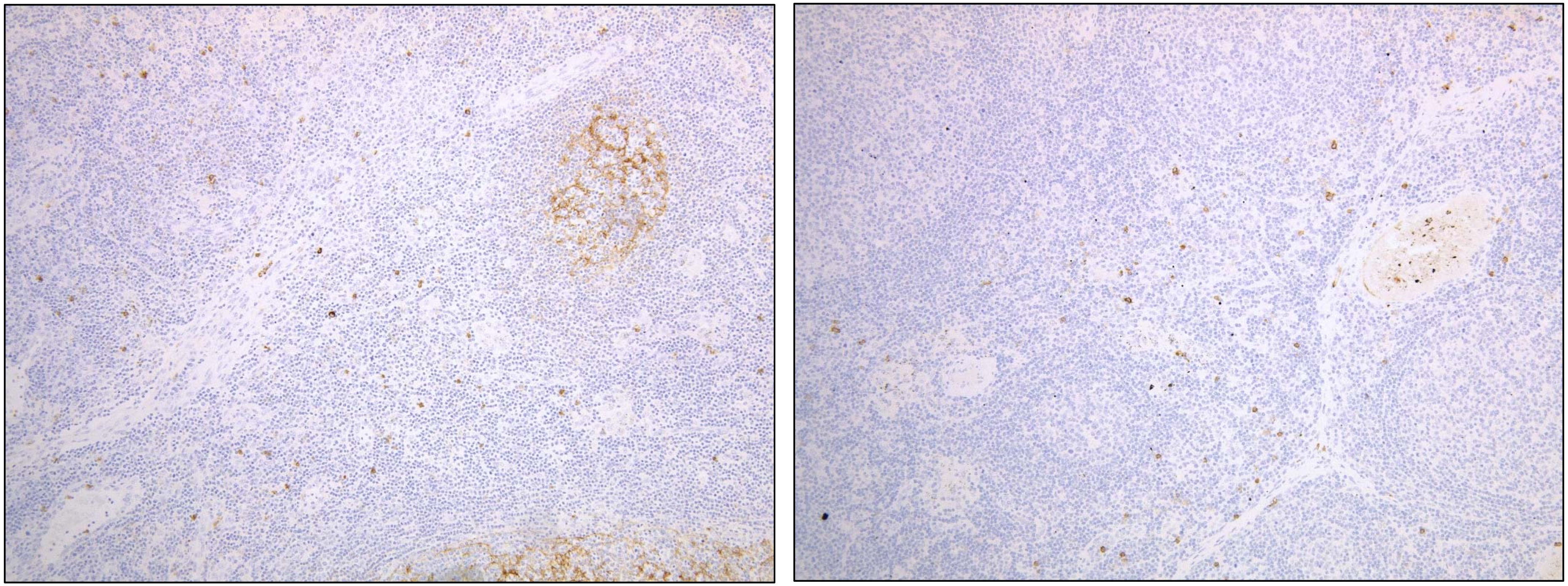

IgE FcεRI

Supplemental Figure 1. Immunoglobulin E (IgE) and IgE receptor (FcεRI) expressing mast cells in non-specific lymphoid hyperplasia.
In low power view, many IgE and FcεRI-positive cells are seen.

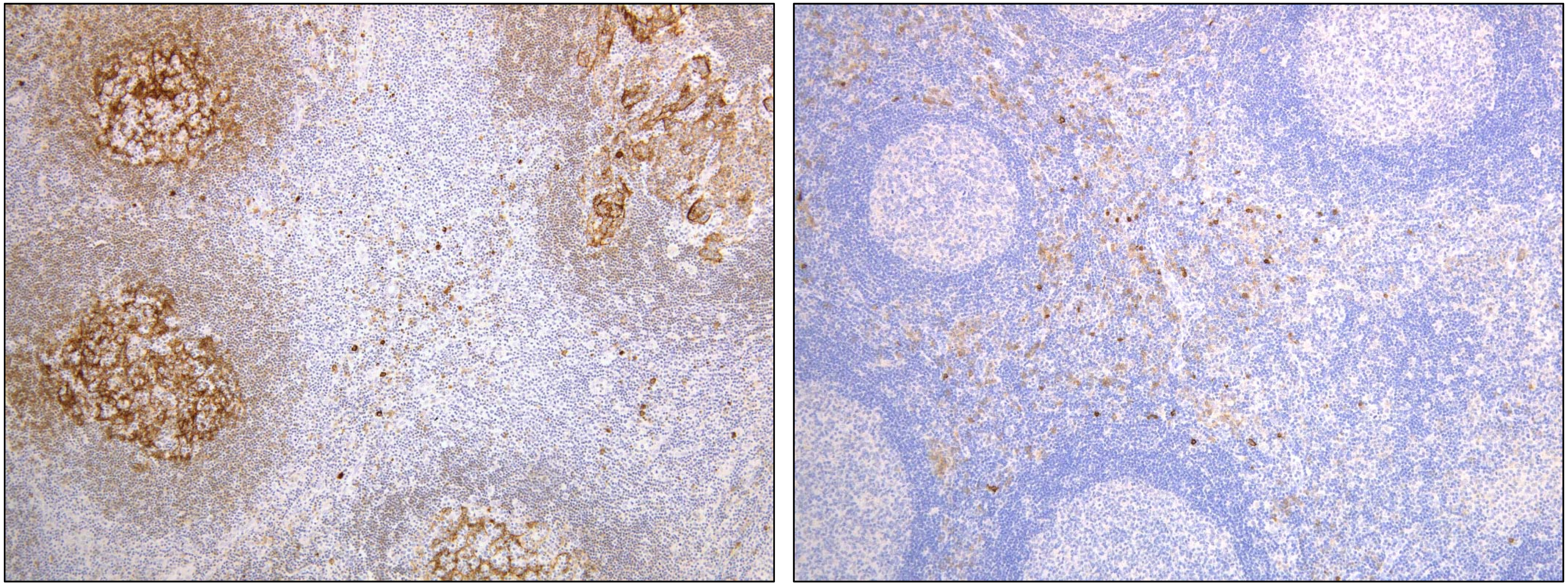

IgE FcεRI

Supplemental Figure 2. Immunoglobulin E (IgE) and IgE receptor (FcεRI) expressing mast cells in IgG4-related lymphadenopathy.

In low power view, many IgE and FcεRI-positive cells are seen.
